# Differential Antihypertensive Effects of Oral Doses of Acetylcholine between Spontaneously Hypertensive Rats and Normotensive Rats

**DOI:** 10.3390/foods10092107

**Published:** 2021-09-06

**Authors:** Shohei Yamaguchi, Kento Matsumoto, Wenhao Wang, Kozo Nakamura

**Affiliations:** 1 Department of Science and Technology, Graduate School of Medicine, Science and Technology, Shinshu University, 8304, Minamiminowa, Nagano 399-4598, Japan; 19hs505d@shinshu-u.ac.jp (S.Y.); 20hs502e@shinshu-u.ac.jp (W.W.); 2 Itoen Co., Ltd., 21, Mekami, Makinohara, Shizuoka 421-0516, Japan; kento-matsumoto@itoen.co.jp; 3 Institute of Agriculture, Academic Assembly, Shinshu University, 8304, Minamiminowa, Nagano 399-4598, Japan

**Keywords:** acetylcholine, blood pressure, functional food, spontaneously hypertensive rats, Wistar–Kyoto rats

## Abstract

Acetylcholine (ACh) is a novel antihypertensive food component. Here, we demonstrate the differential effects of oral ACh on high and normal blood pressure in rats. Spontaneously hypertensive rats (SHRs) and Wistar–Kyoto (WKY) rats were administered ACh orally. The blood pressure and heart rate of SHRs were significantly lowered with ACh doses of 10^−5^ and 10^−3^ mol/kg body weight (b.w.), and the urinary catecholamine levels were significantly decreased with 10^−3^ mol/kg b.w. In contrast, oral ACh administration had no effect on WKY rats. This difference was likely caused by differences in sympathetic nervous activity and the baroreflex between strains. Comparison of gene sequences between the two strains revealed *Chga* mutations, suggesting that changes in the expression of chromogranin A might be involved in the baroreflex in SHRs. Oral ACh had an antihypertensive effect under hypertension but not normotension, indicating that this may be used safely to prevent hypertension.

## 1. Introduction

Hypertension is an important risk factor for cardiovascular disease, accounting for approximately 17 million deaths per year worldwide and corresponding to almost one-third of overall global mortality [1]. In 2015, the number of hypertensive patients in the world was estimated to be 1.13 billion [2]. The World Health Organization advises reducing hypertension to reduce the risk for cerebrocardiovascular diseases and recommends improving dietary habits as a preventive method against hypertension [3]. Antihypertensive foods are expected to prevent and manage hypertension, and various types of food factors with pressure-lowering effects have been studied [4]. Acetylcholine (ACh) is a novel antihypertensive food factor recognized as a functional substance in “Foods with Function Claims” in 2020 [5].

Previously, we isolated choline esters, including ACh and lactoylcholine, from lactofermented foods as active compounds that have an antihypertensive effect at low doses [6,7]. Although choline esters are found widely in various vegetables and fruits, eggplant (*Solanum melongena* L.) contains approximately 3000-times more ACh than other crops [8]. In a randomized placebo-controlled trial comprising individuals with higher blood pressure (BP), the intake of eggplant powder, providing 2.3 mg/day of ACh, improved BP, demonstrating the effectiveness of ACh as a novel antihypertensive food factor [9]. A wide variety of eggplant species contain sufficient levels of ACh to achieve the effective daily intake amount. Additionally, ACh is distributed uniformly in the fruit and is stable under heat [10]. Eggplant is a major agricultural product that is cultivated worldwide, and the antihypertensive effect of ACh is expected to be exploited globally.

Various types of antihypertensive food factors exist; however, there are concerns about the side effects of some of these factors. Angiotensin-converting enzyme (ACE) inhibitory peptides, which are among the most well-known antihypertensive food factors, exert their effects by suppressing the production of angiotensin II, which is associated with increasing BP [11]. ACE inhibition suppresses the degradation of bradykinin, leading to the accumulation of bradykinin in bronchi, which is associated with a dry cough and angioedema. Gamma-aminobutyric acid (GABA) is another well-known antihypertensive substance contained in botanical and fermented foods. However, oral doses of GABA have been associated with adverse effects, such as loss of appetite, diarrhea, and inhibition of emotional incontinence improvement [12]. In addition, overdoses of these compounds can cause hypotension via the excessive inhibition of the enzyme or receptor activation. Hypotension is one of the major side effects of BP-lowering compounds. Antihypertensive drugs are appropriately prescribed by doctors to hypertensive patients to minimize the side effects. In contrast, antihypertensive foods are widely available without a prescription, even to those with normal BP. Therefore, hypotension due to the consumption of antihypertensive food factors should be considered. One clinical trial demonstrated the BP-improving effect of ACh in individuals with higher BP without side effects [9]. However, the effects of oral doses of ACh in individuals with normal BP are currently unknown. Such differences in efficacy based on differences in BP need to be investigated to establish the safety of ACh as a novel antihypertensive food factor. We performed the present study to elucidate the efficacy and safety of oral doses of ACh on rats with normotension.

In this study, we investigated the effects of oral doses of ACh on spontaneously hypertensive rats (SHRs), produced by the selective mating of hypertensive Wistar-strain rats [13] and Wistar–Kyoto (WKY) rats, which have been inbred from the same parental strain and are utilized as a normotensive control for SHRs. We explored the changes in BP, heart rate (HR), and excretion of catecholamine after single oral doses of ACh to determine the differential effects of ACh under hypertension and normotension. Furthermore, we compared gene sequences of SHR and WKY rats to examine potential causes of the differential effects of oral ACh; this is because hypertension in SHRs may be caused by mutations in genes involved in BP regulation in WKY rats [14].

## 2. Materials and Methods

### 2.1. Chemicals

Ultra-pure water, which has a specific resistance of 18.2 MΩ/cm, was produced using an ultra-pure water system (Ariumu 611, Sartorius Co., Göttingen, Germany). Methanol (HPLC-grade), formic acid, 5 mol/L hydrochloric acid, acetic acid, and 2-[4-(2-hydroxyethyl)-1-piperazinyl] ethanesulfonic acid (HEPES) were purchased from Nacalai Tesque, Inc. (Kyoto, Japan). ACh chloride was purchased from Kanto Chemical Co., Inc. (Tokyo, Japan). DL-adrenaline (AD), hydrogen tartrate L-noradrenaline (NAD) monohydrate, and isoproterenol hydrochloride were purchased from Tokyo Chemical Industry Co., Ltd. (Tokyo, Japan).

### 2.2. Animals and Ethics Statement

Male 9-week-old SHR (SHR/Izm) and WKY rats (WKY/Izm) weighing 235.0 ± 6.2 and 248.6 ± 1.6 g, respectively, were purchased from Japan SLC, Inc. (Shizuoka, Japan). Animals were housed individually in metabolic cages (KN-646, Natsume Seisakusho Co., Ltd., Tokyo, Japan). The rats were bred under controlled conditions at 23 ± 4 °C and 50 ± 20% relative humidity under a 12 h light–dark cycle (light period: 7 a.m. to 7 p.m.) with ad libitum access to laboratory food (MF, Oriental yeast Co., Ltd., Tokyo, Japan) and tap water. All experiments were performed with the approval of the Animal Care Committee of the Faculty of Shinshu University (approval number: 290059).

### 2.3. BP Measurement after Oral Administration of ACh

Four SHR and four WKY rats were acclimated in the breeding room for 1 week, and diurnal BP was measured at 9 am, and subsequently after 3, 6, 9, and 24 h for 1 week before the administration test to confirm BP. Four animals of each strain, aged 11–13 weeks, were administered single oral doses of ACh (10^−7^ mol/kg b.w., 10^−5^ mol/kg b.w., and 10^−3^ mol/kg b.w.) using a feeding tube in the morning. Control rats were administered pure water. The rats were fasted after 8 p.m. the day before each administration. The tail-cuff method (BP-98A, Softron Co., Tokyo, Japan) was used to measure the systolic BP (SBP), diastolic BP (DBP), and HR before and 3, 6, 9, and 24 h after drug administration using a previously reported protocol [6].

### 2.4. Urine Sampling after Oral Administration of a Single Dose of ACh

Twelve SHR and 12 WKY rats were acclimated in the breeding room for 1 week and assigned to the ACh (n = 6 per strain) and control (n = 6 per strain) groups. At 12 weeks of age, rats belonging to the ACh group or the control group were given a single oral dose of ACh (10^−3^ mol/kg b.w.) or pure water, respectively, via a feeding tube in the morning. Urine samples were collected from 6 to 12 h after administration for 6 h in a volumetric flask. Thereafter, 250 μL of 5 mol/L hydrochloric acid was added to the flask in advance to prevent the degradation of AD and NAD. The urine samples were stored at −80 °C until analysis.

### 2.5. Quantitation of AD and NAD in Urine Samples by Liquid-Chromatography-Tandem Mass Spectrometry (LC-MS/MS) Analysis

After thawing, the urine samples were centrifuged at 13,374× *g* for 3 min at room temperature (rt, 23 ± 2 °C), and 200 μL of the obtained supernatant was added to a solid-phase extraction column (Mono Spin PBA, GL Sciences Inc., Tokyo, Japan). To condition the column, 200 μL of elution solvent (1% [*v*/*v*] acetic acid) was added to it and the column was centrifuged (10,000× *g*, 1 min, rt); thereafter, 200 μL of wash solvent (10 mmol/L HEPES-NaOH buffer, pH 8.5) was added to the column and centrifuged for 1 min. Subsequently, a mixture of 200 μL of urine sample, 80 μL of binding solvent (1.5 mol/L HEPES-NaOH buffer, pH 8.5), and 20 μL of 500 ng/mL isoproterenol (internal standard) was added to the column and centrifuged (10,000× *g*, 2 min, rt). The filtrate was added again to the column and centrifuged (10,000× *g*, 2 min, rt). Next, 200 μL of the wash solvent was added to the column and centrifuged (10,000× *g*, 1 min, rt); this operation was repeated one more time. Finally, 200 μL of the elution solvent was added to the column and centrifuged (10,000× *g*, 1 min, rt); the eluate was collected as the analytical sample. The AD and NAD levels in the analytical samples were quantitated by LC-MS/MS analysis under the following conditions. The analytes were separated by LC (LC-2040C-3D, Shimadzu Co., Kyoto, Japan) using the YMC-Triart PFP (4.6 × 250 mm, 5 μm), with a mobile water phase containing 15% (*v*/*v*) methanol and 25 mmol/L formic acid, at a flow rate of 0.5 mL/min and column temperature of 40 °C. The mass spectrometer (LCMS-8045, Shimadzu Co., Kyoto, Japan) was operated with electrospray ionization in positive mode, and the analytes were detected in multiple reaction monitoring (MRM) mode. The analytical conditions of MS were capillary voltage of 4.0 kV, interface temperature of 300 °C, desolvation temperature of 350 °C, nebulizer gas flow of 3 L/min, heating gas flow of 10 L/min, and drying gas flow of 10 L/min. The Q1 Prerod bias, collision energy voltage, and Q3 Prerod bias were −13, −18, and −24 V (AD); −17, −18, and −21 V (NAD); and −20, −10, and −20 V (isoproterenol), respectively. The MRM transitions were set as *m/z* 166.05 → 107.15 (AD), *m/z* 152.00 → 107.10 (NAD), *m/z* 212.30 → 194.30 (isoproterenol). Calibration curves were obtained by analyzing standard solutions, and AD and NAD levels in urine samples were calculated using the external standard method. Loss due to pretreatment was corrected based on the recovery of the internal standard. The catecholamine concentration was calculated as sum of the AD and NAD concentrations.

### 2.6. Identification of Single Nucleotide Polymorphisms (SNPs) in Genes Involved in BP Regulation

Genome sequence data for SHR/Izm and WKY/Izm rats were purchased from Disease Model Cooperative Research Association [15]. The Integrative Genomics Viewer browser was used to compare the genome sequences and to estimate genetic differences in the expression and function of proteins involved in BP regulation. SNPs in the *Chga* gene were compared among the SHR/Izm, WKY/Izm, SHR/NCrlCrlj, WKY/NCrlCrlj rats [14], and Brown Norway rats (NCBI GenBank Assembly ID: GCA_000001895.4).

### 2.7. Statistical Analysis

All experimental results are presented as the mean ± standard error, and significant statistical differences were detected at *p* < 0.05 as determined by a Student’s *t*-test. Statistical analyses were conducted using Microsoft^®^ Excel^®^ for Microsoft 365 MSO (16.0.13901.20276).

## 3. Results

### 3.1. General Characteristics of SHRs and WKY Rats Used in the Study

**Figure 1:**
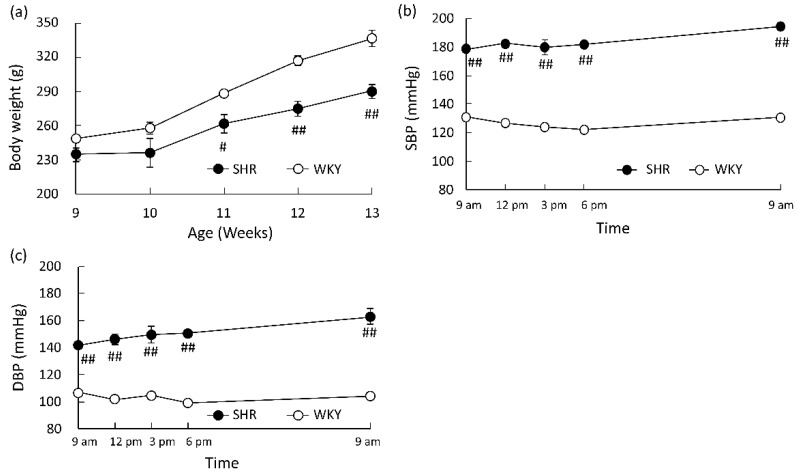
General conditions of SHRs and WKY rats. SBP (**a**) and DBP (**b**) at 9 weeks of age. Body weight at 9–13 weeks of age (**c**). ○: WKY rats (n = 4), ●: SHRs (n = 4). Each data point and error bar represent the mean ± S.E. # *p* < 0.05, ## *p* < 0.01, versus WKY/Izm, as evaluated by a Student’s *t*-test. SBP, systolic blood pressure; DBP, diastolic blood pressure; SHR, spontaneously hypertensive rat; WKY, Wistar–Kyoto.

Changes in the body weight and BP of rats used in this study are shown in Figure 1a–c. The body weights of WKY rats were significantly higher than those of SHRs after the age of 11 weeks. Both the SBP and DBP of SHRs were significantly higher than those of WKY rats throughout the day. The diurnal SBP and DBP of both strains were almost constant, and ranged from 15.3 (178.8–194.2) mmHg for SBP and 20.9 (142.0–162.9) mmHg for DBP in SHRs and 8.8 (121.9–130.8) mmHg for SBP and 7.3 (99.3–106.5) mmHg for DBP in WKY rats.

### 3.2. Effects of Orally Administered ACh on the BP of SHRs and WKY Rats

**Figure 2:**
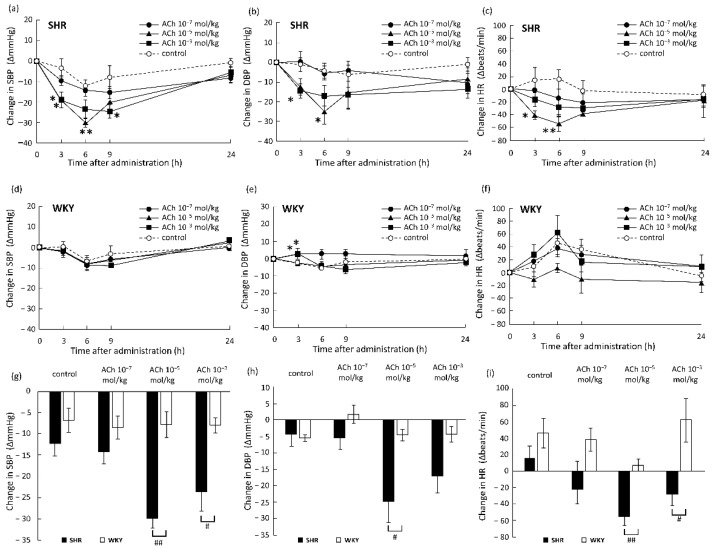
Changes in SBP (**a**,**d**), DBP (**b**,**e**), and HR (**c**,**f**) in SHRs (**a**–**c**) and WKY rats (**d**–**f**) after oral administration of ACh. Pure water was administered as a control. ○: Control (n = 4), ●: 10^−7^ mol/kg b.w. ACh (n = 4), ▲: 10^−5^ mol/kg b.w. ACh (n = 4), ■: 10^−3^ mol/kg b.w. ACh (n = 4). Data at 6 h after administration are summarized to show differences in the effects on SBP (**g**), DBP (**h**), and HR (**i**) between SHRs and WKY rats. ■: SHR (n = 4 in each group), □: WKY rats (n = 4 in each group). Each data point and error bar represent the mean ± S.E. * *p* < 0.05, ** *p* < 0.01, versus control group, # *p* < 0.05, ## *p* < 0.01, versus WKY rats, as evaluated by a Student’s *t*-test. SBP, systolic blood pressure; DBP, diastolic blood pressure; HR, heat rate; ACh, acetylcholine; SHR, spontaneously hypertensive rat; WKY, Wistar–Kyoto; b.w., body weight.

The effects of oral doses of ACh on the BP (SBP and DBP) and HR in SHRs are shown in Figure 2a–c. An oral dose of ACh at 10^−7^ mol/kg b.w. caused no significant changes in the SBP, DBP, and HR. An oral dose of ACh at 10^−5^ mol/kg b.w. significantly reduced the SBP and HR at 3 and 6 h and significantly reduced the DBP at 6 h after administration compared to those in the control group. An oral dose of 10^−3^ mol/kg b.w. of ACh significantly reduced SBP at 3 and 9 h, and significantly reduced DBP at 3 h after administration; however, there was no significant change in HR. These results demonstrated that oral administration of a single dose of ACh greater than 10^−5^ mol/kg b.w. had acute BP- and HR-lowering effects in SHRs. The effects of oral doses of ACh on the BP and HR of WKY rats are shown in Figure 2d–f. The DBP significantly increased 3 h after oral doses of 10^−7^ and 10^−3^ mol/kg b.w. of ACh, whereas the SBP and HR were not significantly changed. No significant changes were observed in SBP, DBP, and HR with an oral dose of 10^−5^ mol/kg b.w. of ACh.

Differences in the effects of oral doses of ACh on SBP, DBP, and HR 6 h after administration between SHRs and WKY rats are summarized in Figure 2g–i. In the control group, changes in SBP, DBP, and HR did not differ significantly between SHR and WKY rats. Oral doses of ACh induced a greater reduction in BP and HR in SHRs than in WKY rats (SBP [−22.1 mmHg, *p* < 0.01], DBP [−20.4 mmHg, *p* < 0.05], and HR [−61.3 beats/min, *p* < 0.01] at a dose of 10^−5^ mol/kg b.w., and SBP [−15.5 mmHg, *p* < 0.05] and HR [−89.9 beats/min, *p* < 0.05] at a dose of 10^−3^ mol/kg b.w.).

### 3.3. Effects of Orally Administered ACh on AD and NAD Excretion in SHRs and WKY Rats

**Figure 3:**
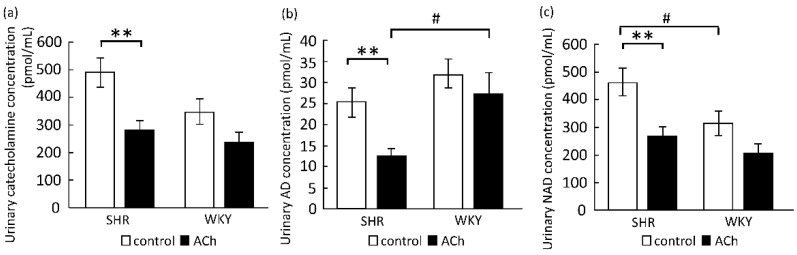
Excretion of catecholamine (**a**), AD (**b**), and NAD (**c**) in urine samples collected from SHR and WKY rats 6–12 h after oral administration of ACh (10^−3^ mol/kg b.w.) or pure water (control). □: Control (n = 6). ■: ACh 10^−3^ mol/kg b.w. (n = 6). The catecholamine concentration was calculated as sum of the concentrations of AD and NAD. Each data point and error bar represent the mean ± S.E. ** *p* < 0.01, versus the control group, # *p* < 0.05, versus WKY rats, as evaluated by a Student’s *t*-test. AD, adrenaline; NAD, noradrenaline; ACh, acetylcholine; SHR, spontaneously hypertensive rat; WKY, Wistar–Kyoto; b.w., body weight.

The catecholamine concentrations in the urine samples collected 6–12 h after the oral administration of ACh at 10^−3^ mol/kg b.w. for 6 h, representing the period of maximum BP-lowering effect, are shown in Figure 3a–c. The urinary catecholamine concentration was significantly low in the ACh group of SHRs, but not in WKY rats. The AD and NAD levels are shown in Figure 3b,c, respectively. The urinary NAD level in SHRs was higher than that in WKY rats in the control group. The oral dose of ACh significantly reduced both urinary AD and NAD levels in SHRs but not in WKY rats compared to those in the control group. The urinary AD level in WKY rats was significantly higher than that in SHRs in the ACh group (*p* = 0.0179).

### 3.4. Mutations in the BP Regulation-Related Chga Gene in SHRs and WKY Rats

The complete genome sequences of the SHR (SHR/Izm) and WKY (WKY/Izm) strains used in this study and that of Brown Norway rats were compared to identify mutated genes involved in the regulation of BP. Consequently, the *Chga* gene (NM 021655), which encodes chromogranin A, was identified. Chromogranin A has been reported to be involved in the regulation of sympathetic transmission and the baroreflex [16]. SNPs in the *Chga* gene are shown in Table 1. In SHRs, there were substitutions in the proximal promoter region and exon 8. In WKY rats, there were substitutions in the proximal promoter region and in exons 7 and 8. Three mutations (A-1600T, C-161T, and G+11184T) were shared between SHRs and WKY rats, and the C-43T mutation was unique to SHRs when compared to the sequence in WKY and Brown Norway rats. The mutation in exon 7 in WKY rats was silent. The mutation in exon 8 was located in the 3′-untranslated region of the mRNA encoding chromogranin A. No mutation that caused an amino acid substitution in chromogranin A was identified. Mutations in introns are shown in Table S1; SHRs possessed more intronic mutations than WKY rats. As shown in column NCrlCrlj in Table 1, four mutations (A-1600T, C-161T, C-43T, and G+11184T) were observed in SHR/NCrlCrlj rats at a similar location in the *Chga* gene; however, these were not identified in WKY/NCrlCrlj rats in a previous study [14].

## 4. Discussion

Choline esters, including ACh, contained in various agricultural products, are novel functional food components first isolated as antihypertensive compounds from lactofermented foods [6,7,8]. A single oral dose of eggplant powder equivalent to 10^−9^ mol/kg b.w. of ACh was found to significantly lower the BP in SHRs by acting on the M3 muscarinic ACh receptor [17]. The effective dose varied from that in the present study because the SHR/NCrlCrlj line used was obtained from a different closed colony than the one from which the SHR/Izm line used in the present study was obtained. Moreover, the consumption of eggplant powder containing 2.3 mg of ACh was shown to improve BP in subjects with higher BP in a randomized controlled trial [9]. These studies demonstrated the efficacy of ACh as an antihypertensive food factor. In the present study, an antihypertensive effect of oral doses of ACh was observed in hypertensive, but not in normotensive, rats. Differences in the BP-lowering effect of oral ACh between SHRs and WKY rats are discussed later in this section.

In our previous study, the antihypertensive effect of eggplant powder rich in ACh on SHRs was suggested to be caused by a decrease in NAD and AD levels accompanied by an attenuation of sympathetic nervous activity [17]. NAD and AD are agonists of the sympathetic nervous system, and directly increase BP through vasoconstriction and increased cardiac output via adrenaline receptors. Sympathetic stimulation induces the secretion of NAD into the synaptic cleft from the sympathetic nerve terminal, and the secretion of AD and NAD into the blood from the adrenal medulla. Most NAD released from the nerve terminal is absorbed into the presynaptic terminal by NAD transporters for reuse; however, some NAD leaks into the blood and is excreted in the urine. Therefore, the urinary levels of catecholamine comprising NAD and AD were indicative of sympathetic nervous activity for the duration of the experiment. In the present study, the higher level of NAD in SHRs compared to that in WKY rats was due to excessive sympathetic nerve activity, with NAD and AD released from the adrenal glands of SHRs [18,19]. The significant decrease in the urinary NAD and AD levels in SHRs following the ingestion of ACh indicated that oral ACh attenuated the sympathetic nervous activity, reduced the AD and NAD release, and lowered the BP. The significant reduction in HR supported the sympathetic activity in SHRs in the ACh group. Our previously proposed mechanism for the antihypertensive effect of ACh, specifically that ACh acts on gastrointestinal M3 muscarinic ACh receptors, suppresses the sympathetic nervous activity, and lowers the BP [17], supported the effects on the BP, HR, and urinary catecholamines observed in the present study.

In contrast, oral doses of ACh did not lower the BP or decrease the urinary AD and NAD levels in WKY rats via sympathetic attenuation. The difference in the effects of oral ACh on BP between the two strains is assumed to be due to differences in the reduction of AD and NAD levels, which are regulated by sympathetic nervous activity. The AD level in WKY rats was significantly higher than that in SHRs in the ACh groups, although the AD levels in the control group were similar between the strains. This difference may be responsible for the sympathetic nerve function, including the baroreflex, in each strain. The *Chga* gene sequence differed between SHR/Izm and WKY/Izm rats in this study. Mutations of the *Chga* gene in the SHR/Izm line were similar to those in the SHR/NCrlCrlj line. Notably, the amino acid sequence of chromogranin A was the same in SHR/Izm and WKY/Izm, indicating that no functional alteration had occurred. However, a difference in the *Chga* gene promoter was observed in SHR strains, which could have affected the expression of chromogranin A. The genetic mutation in the proximal promoter of *Chga* in SHRs was suggested to contribute to the development of hypertension due to the modulation of sympathetic nervous activity and baroreflex function [14]. In *Chga*-knockout mice, BP is increased and baroreflex function is decreased [20,21]. The baroreflex is a homeostatic system that regulates the circulation. An increase or decrease in BP induces reflex responses to decrease or increase the sympathetic outflow via the baroreceptor on the carotid sinus or aortic arch to minimize fluctuations in BP [22]. The baroreflex responsible for lowering the BP increases the sympathetic outflow in the adrenal sympathetic nerve, resulting in the secretion of catecholamines and an increase in BP [23]. Therefore, it was hypothesized that the higher AD levels in the urine of WKY rats following oral administration of ACh was the result of a baroreflex response acting to restore the lowered BP. According to this hypothesis, the elevated DBP in WKY rats 3 h after the oral dose of ACh could be due to the baroreflex. Additionally, the baroreflex function of SHRs is deteriorated to a greater extent than in normotensive rats [24]. It has also been hypothesized that the lowered BP and HR induced by oral doses of ACh in SHRs could not be restored because of the functional decline in the baroreflex.

The results of the present study show that oral ACh does not exert a BP-lowering effect under normotension but exerts a significant BP-lowering effect under hypertension. Similarly, GABA has been reported to show BP-lowering effects in SHRs but not in WKY rats [25]. GABA has been proposed to exert its effects by directly suppressing the sympathetic nervous activity via GABA_B_ receptors [26]. ACh and GABA have similar BP-lowering mechanisms, which include the attenuation of sympathetic nervous activity, although the site of action differs. This hypothesis for the mechanism underlying the effects of ACh may explain the differences in the BP-lowering effects of GABA between rat strains. Sympathetic nervous activity and baroreflex function are positively and negatively correlated with BP, respectively, in individuals with severe hypertension [27,28]. In our clinical study, the BP-improving effect mediated by the ingestion of 2.3 mg/day ACh was higher in the grade 1 hypertension group than in the normal–high BP group [9], suggesting that oral doses of ACh might be more effective for individuals with higher BP. An oral dose of ACh is not expected to lower the BP in individuals with normal BP and normal baroreflex function. Thus, ACh is predicted to act as a low-dose antihypertensive food factor with high safety, which could be used to prevent or improve hypertension, without an effect on normotensive individuals. In individuals with normal–high BP, the BP-improving effect could be weakened by the baroreflex function. In our clinical study, the effect of eggplant-derived ACh was mild in individuals with normal–high BP, which is believed to be caused by the endogenous factor of the baroreflex, and the exogenous factor of a low outside temperature [9]. Moreover, a deteriorated baroreflex is likely to be involved in enhancing the BP-lowering effects in a hypertensive state caused by other antihypertensive compounds, including depressors.

In conclusion, we demonstrate that oral administration of ACh has an antihypertensive effect, causing a reduction in catecholamine levels in hypertensive rats but not in normotensive rats. The antihypertensive effect is probably caused by suppression of the sympathetic nervous activity. In SHRs, in which the increased sympathetic nervous activity contributed to hypertension, the lowering of BP due to the reduction in catecholamine activity was distinguished. In addition, the BP-lowering status was possibly maintained by the deteriorated baroreflex function in SHRs. The dependence of the specificity of the antihypertensive effect on the BP status indicates the safety of ACh as a novel antihypertensive food component. Foods rich in ACh, such as eggplant and fermented foods, are useful for the management of hypertension through diet. To test our hypothesis, in the future, we plan to further investigate the effect of oral doses of ACh on the sympathetic nervous activity, including the baroreflex, via direct measurements of the sympathetic nervous activity.

## Figures and Tables

**Table 1:** Single nucleotide polymorphisms (SNPs) in the *Chga* gene between WKY rats (WKY/Izm) and SHRs (SHR/Izm) using Brown Norway rats as a reference genome.

Strain Alleles								
	Izm				NCrlCrlj [14]			
Domain	Location	BN	WKY	SHR	Location	BN	WKY	SHR
Promoter	A-1600T	A	T	T	A-1616T	A	A	T
Promoter	C-161T	C	T	T	C-177T	C	C	T
Promoter	C-43T	C	C	T	C-59T	C	C	T
Exon 7	C+8850T	C	T	C	n/a			
Exon 8	G+11184T	G	T	T	G+11177T	G	G	T

n/a: The substitution corresponding to the C+8850T mutation has not been reported previously. SNP, single nucleotide polymorphism; BN, Brown Norway; SHR, spontaneously hypertensive rat; WKY, Wistar–Kyoto; *Chga*, gene encoding chromogranin A.

## Data Availability

The data presented in this study are available on request from the corresponding author.

## Supplementary Materials

The following are available online at https://www.mdpi.com/article/10.3390/foods10092107/s1, Table S1: SNPs in introns of the *Chga* gene in WKY rats (WKY/Izm) and SHRs (SHR/Izm) using Brown Norway rats as a reference genome.

## References

[1] World Health Organization (2013). A global Brief on Hypertension: Silent Killer, Global Public Health Crisis. https://www.who.int/cardiovascular_diseases/publications/global_brief_hypertension/en/.

[2] Zhou B., Bentham J., Di Cesare M., Bixby H., Danaei G., Cowan M.J., Paciorek C.J., Singh G., Hajifathalian K., Bennett J.E. (2017). Worldwide trends in blood pressure from 1975 to 2015: A pooled analysis of 1479 population-based measurement studies with 19·1 million participants. Lancet.

[3] World Health Organization (2019). Hypertension. https://www.who.int/news-room/fact-sheets/detail/hypertension.

[4] Iwatani S., Yamamoto N. (2019). Functional food products in Japan: A review. Food Sci. Hum. Well..

[5] Consumer Affairs Agency The System of “Foods with Function Claims” Has Been Launched! 2015. https://www.caa.go.jp/policies/policy/food_labeling/information/pamphlets/pdf/151224_2.pdf.

[6] Nakamura K., Naramoto K., Koyama M. (2013). Blood-pressure-lowering effect of fermented buckwheat sprouts in spontaneously hypertensive rats. J. Funct. Foods.

[7] Nakamura K., Okitsu S., Ishida R., Tian S., Igari N., Yoshihiko A. (2015). Identification of natural lactoylcholine in lactic acid bacteria-fermented food. Food Chem..

[8] Wang W., Yamaguchi S., Koyama M., Tian S., Ino A., Miyatake K., Nakamura K. (2020). LC–MS/MS analysis of choline compounds in Japanese-cultivated vegetables and fruits. Foods.

[9] Nishimura M., Suzuki M., Takahashi R., Yamaguchi S., Tsubaki K., Fujita T., Nishimura J., Nakamura K. (2019). Daily ingestion of eggplant powder improves blood pressure and psychological state in stressed individuals: A randomized placebo-controlled study. Nutrients.

[10] Wang W., Yamaguchi S., Suzuki A., Wagu N., Koyama M., Takahashi A., Takada R., Miyatake K., Nakamura K. (2021). Investigation of the distribution and content of acetylcholine, a novel functional compound in eggplant. Foods.

[11] Iwaniak A., Minkiewicz P., Darewicz M. (2014). Food-originating ACE inhibitors, including antihypertensive peptides, as preventive food components in blood pressure reduction. Compr. Rev. Food Sci. Food Saf..

[12] Alfresa Pharma Co. Interview Form of Gammalon®. 2019, in Japanese. https://file.wuxuwang.com/jpyaopin/530258_2190004F1034_2_001_1F.pdf.

[13] Okamoto K., Aoki K. (1963). Development of a strain of spontaneously hypertensive rats. Jpn. Circ. J..

[14] Friese R.S., Altshuler A.E., Zhang K., Miramontes-Gonzalez J.P., Hightower C.M., Jirout M.L., Salem R.M., Gayen J.R., Mahapatra N.R. (2013). MicroRNA-22 and promoter motif polymorphisms at the Chga locus in genetic hypertension: Functional and therapeutic implications for gene expression and the pathogenesis of hypertension. Hum. Mol. Genet..

[15] Isomura M., Saar K., Ohara H., Hübner N., Kato N., Nabika T. (2015). 1A.05: Comparison of the whole genome sequence revealed genetically distinct loci between SHR/Izm and SHRSP/Izm. J. Hypertens..

[16] Mahata S.K., Mahata M., Fung M.M., O’Connor D.T. (2010). Reprint of: Catestatin: A multifunctional peptide from chromogranin A. Regul. Pept..

[17] Yamaguchi S., Matsumoto K., Koyama M., Tian S., Watanabe M., Takahashi A., Miyatake K., Nakamura K. (2019). Antihypertensive effects of orally administered eggplant (*Solanum melongena*) rich in acetylcholine on spontaneously hypertensive rats. Food Chem..

[18] Judy W.V., Watanabe A.M., Henry D.P., Besch H.R., Murphy W.R., Hockel G.M. (1976). Sympathetic nerve activity: Role in regulation of blood pressure in the spontaenously hypertensive rat. Circ. Res..

[19] Lim D.Y., Jang S.J., Park D.G. (2002). Comparison of catecholamine release in the isolated adrenal glands of SHR and WKY rats. Auton. Autacoid. Pharmacol..

[20] Mahapatra N.R., O’Connor D.T., Vaingankar S.M., Hikim A.P., Mahata M., Ray S., Staite E., Wu H., Gu Y., Dalton N. (2005). Hypertension from targeted ablation of chromogranin A can be rescued by the human ortholog. J. Clin. Investig..

[21] Gayen J.R., Gu Y., O’Connor D.T., Mahata S.K. (2009). Global disturbances in autonomic function yield cardiovascular instability and hypertension in the chromogranin a null mouse. Endocrinology.

[22] Wehrwein E.A., Joyner M.J. (2013). Regulation of blood pressure by the arterial baroreflex and autonomic nervous system. Handb. Clin. Neurol..

[23] Scislo T.J., Augustyniak R.A., O’Leary D.S. (1998). Differential arterial baroreflex regulation of renal, lumbar, and adrenal sympathetic nerve activity in the rat. Am. J. Physiol..

[24] Head G.A., Adams M.A. (1992). Characterization of the baroreceptor heart rate reflex during development in spontaneously hypertensive rats. Clin. Exp. Pharmacol. Physiol..

[25] Hayakawa K., Kimura M., Kasaha K., Matsumoto K., Sansawa H., Yamori Y. (2004). Effect of a gamma-aminobutyric acid-enriched dairy product on the blood pressure of spontaneously hypertensive and normotensive Wistar-Kyoto rats. Br. J. Nutr..

[26] Kimura M., Hayakawa K., Sansawa H. (2002). Involvement of gamma-aminobutyric acid (GABA) B receptors in the hypotensive effect of systemically administered GABA in spontaneously hypertensive rats. Jpn. J. Pharmacol..

[27] Mussalo H., Vanninen E., Ikäheimo R., Laitinen T., Laakso M., Länsimies E., Hartikainen J. (2002). Baroreflex sensitivity in essential and secondary hypertension. Clin. Auton. Res..

[28] Philipp T.H., Distler A., Cordes U. (1978). Sympathetic nervous system and blood-pressure control in essential hypertension. Lancet.

