# Supplementary material for: Differential Antihypertensive Effects of Oral Doses of Acetylcholine between Spontaneously Hypertensive Rats and Normotensive Rats
Source: Foods. 2021 Sep 6;10(9):2107. doi: 10.3390/foods10092107 (PMC8469729; doi:10.3390/foods10092107)
Supplement: Supplementary file 1 [file foods-10-02107-s001.zip › foods-1343394-supplementary.pdf]

Supplementary data

Table S1. SNPs in introns of the *Chga* gene in WKY rats (WKY/Izm) and SHRs (SHR/Izm) using Brown Norway rats as a reference genome.

Strain alleles				
Domain	Location	BN	WKY/Izm	SHR/Izm
Intron2	A+1128G	A	A	G
Intron2	A+1211T	A	A	T
Intron2	T+2078C	T	C	C
Intron2	T+2172G	T	T	G
Intron2	G+2185A	G	G	A
Intron2	C+2642T	C	C	T
Intron2	C+2717T	C	T	T
Intron2	G+2917C	G	C	C
Intron2	G+3048T	G	T	G
Intron2	C+3183T	C	T	n/a
Intron3	T+3976C	T	T	C
Intron4	T+4499C	T	C	C
Intron4	G+4554A	G	A	A
Intron5	C+6596T	C	C	T
Intron6	A+8397G	A	G	G
Intron6	G+8645A	G	A	G
Intron7	G+9430A	G	G	A
Intron7	A+9455G	A	G	G
Intron7	C+9532T	C	T	T
Intron7	G+10889A	G	A/G	A

In the G+10889A mutation in WKY rats, G and A were detected at the same ratio.

SNP, single nucleotide polymorphism; CHGA, chromogranin A; BN, Brown Norway rats; SHR, spontaneously hypertensive rat; WKY, Wistar-Kyoto; n/a, the base corresponding to the location was not available.
